# Inflammatory Markers Predict Survival in Patients With Advanced Gastric and Colorectal Cancers Receiving Anti–PD-1 Therapy

**DOI:** 10.3389/fcell.2021.638312

**Published:** 2021-03-15

**Authors:** Xiaona Fan, Dan Wang, Wenjing Zhang, Jinshuang Liu, Chao Liu, Qingwei Li, Zhigang Ma, Hengzhen Li, Xin Guan, Yibing Bai, Jiani Yang, Changjie Lou, Xiaobo Li, Guangyu Wang, Zhiwei Li

**Affiliations:** ^1^Department of Gastrointestinal Medical Oncology, Harbin Medical University Cancer Hospital, Harbin, China; ^2^Translational Medicine Research and Cooperation Center of Northern China, Heilongjiang Academy of Medical Sciences, Harbin, China

**Keywords:** anti–PD-1 therapy, inflammatory biomarker, advanced gastric and colorectal cancer, response, PFS

## Abstract

There is a lack of useful biomarkers for predicting the efficacy of anti–programmed death-1 (PD-1) therapy for advanced gastric and colorectal cancer. To address this issue, in this study we investigated the correlation between inflammatory marker expression and survival in patients with advanced gastric and colorectal cancer. Data for 111 patients with advanced gastric and colorectal cancer treated with anti–PD-1 regimens were retrospectively analyzed. Neutrophil-to-lymphocyte ratio (NLR), monocyte-to-lymphocyte ratio (MLR), platelet-to-lymphocyte ratio (PLR), and clinical characteristics of each patient were selected as the main variables. Overall response rate, disease control rate, and progression-free survival were primary endpoints, and overall survival and immune-related adverse events (irAEs) were secondary endpoints. The chi-squared test and Fisher’s exact test were used to evaluate relationships between categorical variables. Uni- and multivariate Cox regression analyses were performed, and median progression-free survival and overall survival were estimated with the Kaplan–Meier method. The overall response rate and disease control rate of anti–PD-1therapy in advanced gastric and colorectal tumors were 12.61 and 66.66%, respectively. The patients with MLR < 0.31, NLR < 5, and PLR < 135 had a significantly higher disease control rate than those with MLR > 0.31, NLR > 5, and PLR > 135 (*P* < 0.05). The multivariate analysis revealed that MLR < 0.31, BMI > 18.5, and anti–PD-1 therapy in first-line were associated with prolonged PFS. MLR < 0.31 and BMI > 18.5 were associated with prolonged overall survival. The irAE rate differed significantly between PLR groups, and PLR < 135 was associated with an increased rate of irAEs (*P* = 0.028). These results indicate that the inflammatory markers NLR, MLR, and PLR have clinical utility for predicting survival or risk of irAEs in patients with advanced gastric cancer and colorectal cancer.

## Introduction

Colorectal cancer (CRC) and gastric cancer (GC) rank first and fourth, respectively, in terms of incidence among digestive tract tumors (Siegel et al., 2020). Immune checkpoint inhibitors (ICIs) such as anti–programmed death-1 (PD-1) antibodies have dramatically altered the treatment landscape for several advanced malignancies. For example, anti–PD-1 monoclonal antibody has been shown to confer a survival advantage to patients with gastrointestinal tumors and is now a standard treatment.

Despite observable and lasting responses in many cases, not all cancer patients benefit from immunotherapy. The overall response rate (ORR) of GC to immunotherapy is approximately 11% (Kang et al., 2017; Fuchs et al., 2018). Microsatellite instability (MSI) status is the main predictor of whether patients with CRC will benefit from immunotherapy. However, in the Checkmate-016 trial, the ORR for MSI-high CRC patients was just 31.1% (95% confidence interval [CI]: 20.8-42.9). Responses to immunotherapy vary markedly, and there are currently no reliable predictive markers for selecting patients who are most likely to benefit from a treatment (Pavan et al., 2019). Therefore, there is an urgent need to identify useful and reliable biomarkers for routine clinical use.

Tumorigenesis and tumor progression are closely related to inflammation, as inflammatory cells promote cancer cell proliferation, angiogenesis, and tumor invasion and even influence the efficacy of some anticancer drugs (Qian et al., 2019). As such, inflammatory factors can potentially serve as biomarkers for predicting tumor recurrence and prognosis. The neutrophil-to-lymphocyte ratio (NLR), monocyte-to-lymphocyte ratio (MLR), and platelet-to-lymphocyte ratio (PLR) are prognostic biomarkers (Chen et al., 2019; Imamura et al., 2019; Łochowski et al., 2019; Yang et al., 2019) that have been used to predict response to anti–PD-1 therapy in non-small cell lung cancer (NSCLC), renal cancer, and ovarian cancer, among other malignancies (Baert et al., 2018; Bilen et al., 2018; Cao et al., 2018; Bartlett et al., 2020). In the present study, we analyzed the correlation between NLR, MLR, and PLR and ORR, disease control rate (DCR), and progression-free survival (PFS) as primary endpoints and overall survival (OS) and immune-related adverse events (irAEs) as secondary endpoints in patients with advanced GC and CRC receiving anti–PD-1 therapy in order to determine whether these inflammatory markers have prognostic value in these cancer types.

## Materials and Methods

### Study Design and Objectives

Patients with advanced GC or CRC with unknown MSI status who had received PD-1 inhibitor therapy at Cancer Hospital of Harbin Medical University between September 1, 2018 and July 10, 2020 were included in this retrospective cohort study. The pathologic type of all patients was adenocarcinoma with or without mucinous adenocarcinoma. The last follow-up date was October 27, 2020. Data for patients who received at least two infusions of drug and underwent peripheral blood examination within 2 weeks prior to treatment were analyzed. The samples were promptly centrifuged and processed within 2 h. The cell counting of peripheral blood was measured using the SYSMEX XN-9000 full-automated hematology analyzer (Sysmex, Tokyo, Japan).

Patients who received other anti-tumor therapy within 4 weeks before anti–PD-1 treatment to reduce the influence of previous treatments on peripheral blood index were excluded. Data obtained from electronic medical records and pharmacy databases included patient demographic information and clinical data, hematologic and biochemical parameters at baseline (before the first treatment cycle), concomitant treatments, treatment response, date of last follow-up, and date of death. To eliminate the influence of immunotherapy pseudo-progression, we selected ORR and DCR after 12 weeks of treatment as well as PFS as our primary endpoints; OS and irAE were secondary endpoints.

Progression-free survival was defined as the time from the first treatment cycle with anti–PD-1 agent to radiographically recorded disease progression or death (event) or the last follow-up (censored). OS was defined as the time from the first treatment with anti–PD-1 agent to death, or was censored at the date of last patient contact. To evaluate treatment response, scheduled computed tomography or magnetic resonance imaging was performed every 3 months according to RECIST 1.1 criteria or with clinical worsening of the patient’s condition. ORR was defined as the ratio of the sum of complete response (CR) plus partial response (PR). DCR was defined as the ratio of the sum of CR and PR and stable disease (SD). Treatment was continued until confirmation of disease progression, unacceptable toxicity, or voluntary termination of treatment. The vast majority of patients received anti–PD-1 preparation combined with chemotherapy, radiotherapy, or targeted therapy. This study was approved by the Ethics Review Board of the Cancer Hospital of Harbin Medical University.

### Statistical Analysis

Descriptive statistics were used to summarize patients’ demographic and clinical data and treatment information. NLR was calculated as absolute neutrophil count/total lymphocyte count; MLR was calculated as monocyte count/total lymphocyte count; and PLR was calculated as platelet count/total lymphocyte count. The cutoff value for NLR was 5 in accordance with previous studies (Kartolo et al., 2020; Ueda et al., 2020); the optimal cutoff values for MLR and PLR were 0.31 and 135, respectively, which were determined using R-4.0.2 software (R Core Team, 2014). The chi-squared test and Fisher’s exact test were used to assess relationships among categorical variables. Cox proportional hazard models were used to evaluate the relationship between each variable and disease progression and patient survival, and median (m) PFS and median (m) OS were estimated by the Kaplan–Meier method. Statistical differences between each variable and the probability of mPFS and mOS were analyzed with the log-rank test; Cox multivariate regression analysis was performed via forward LR method; the results are expressed as hazard ratio (HR) 95% CI, and *P* values <0.05 were considered statistically significant. Statistical analyses were performed using SPSS v23.0 (IBM, Armonk, NY, United States) and R software programs.

## Results

### Patient Characteristics

Patient demographic information and disease characteristics are presented in Table 1. A total of 111 patients with advanced GC or CRC treated with anti-PD-1therapy were enrolled in this study, including 55 women and (49.55%) and 56 men (50.45%). There were 23 patients (20.72%) aged ≥65 years and 88 (79.28%) aged <65 years; There were no differences between any inflammatory marker group and liver, lung, or peritoneal metastasis at baseline; There were no differences between any inflammatory marker and concomitant treatment including chemotherapy, targeted therapy, and radiotherapy. However, statistically significant differences were found between MLR and treatment line (*P* = 0.035).

**TABLE 1:** Characteristics of patients in this study.

		NLR			PLR			MLR		
				
Characteristic	Total (*N* = 111) *N* (%)	<5.0 (*n* = 94, 84.7%)	≥5.0 (*n* = 17, 15.3%)	*P* value	<135 (*n* = 55, 49.5%)	≥135 (*n* = 56, 50.5%)	*P* value	<0.31 (*n* = 61, 55.0%)	>0.31 (*n* = 50, 45.0%)	*P* value
**Sex**										
Female	55 (49.5%)	47	8	0.823	31	28	0.502	28	27	0.396
Male	56 (50.5%)	47	9		24	28		33	23	
**Age**										
≥ 65	23 (20.7%)	20	3	0.767	12	11	0.777	12	11	0.763
< 65	88 (79.2%)	74	14		43	45		49	39	
**Cancer type**										
Gastric cancer	50 (42.6%)	45	5	0.159	21	29	0.150	28	22	0.841
Colorectal cancer	61 (57.6%)	49	12		34	27		33	28	
**Treatment line**										
First line	51 (45.9%)	46	5	0.137	29	22	0.155	34	17	0.035
> First line	60 (54.1%)	48	12		26	34		27	33	
**Liver metastasis**										
No	55 (49.5%)	49	6	0.210	27	28	0.924	34	21	0.150
Yes	56 (50.5%)	45	11		28	28		27	29	
**Lung metastasis**										
No	84 (75.6%)	70	14	0.486	43	41	0.542	48	36	0.414
Yes	27 (24.4%)	24	3		12	15		13	14	
**Peritoneal metastasis**										
No	90 (81.1%)	76	14	0.884	48	42	0.099	51	39	0.453
Yes	21 (18.9%)	18	3		7	14		10	11	
**With chemotherapy**										
No	37 (33.3%)	30	7	0.456	19	18	0.788	17	20	0.177
Yes	74 (66.7%)	64	10		36	38		44	30	
**With Targeted therapy**										
No	67 (60.4%)	60	7	0.079	36	31	0.277	41	26	0.103
Yes	44 (39.6%)	34	10		19	25		20	24	
**With Radiotherapy**										
No	104 (93.7%)	89	15	0.314	51	53	0.678	58	46	0.506
Yes	7 (6.3%)	5	2		4	3		3	4	

**P* values in bold indicate statistically significant differences (*P* < 0.05).*

*MLR, monocyte-to-lymphocyte ratio; NLR, neutrophil-to-lymphocyte ratio; PLR, platelet-to-lymphocyte ratio.*

### Treatment Response

**FIGURE 1:**
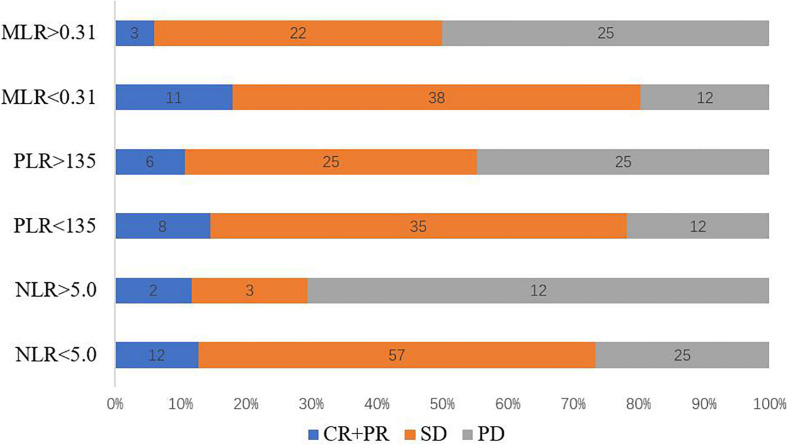
Response rates corresponding to baseline NLR, PLR, and MLR. CR, complete response; MLR, monocyte-to-lymphocyte ratio; NLR, neutrophil-to-lymphocyte ratio; PLR, platelet-to-lymphocyte ratio; PR, partial response; SD, stable disease.

Of the 111 patients with stage IV GC and CRC who received anti–PD-1 inhibitor therapy, DCR at 12 weeks was 66.66% and ORR was 12.61% (Figure 1). The patients with MLR < 0.31 had the highest ORR of 18.03%, whereas patients with MLR > 5 had the lowest ORR of only 6.0% (*P* = 0.057). There was no significant inter-group differences in the ORR (NLR < 5 vs. NLR > 5, *P* = 0.909, PLR < 135 vs. PLR > 135, *P* = 0.543). The patients with MLR < 0.31 had DCR of 79.66%, whereas patients with MLR > 5 had ORR of only 51.92%. Moreover, significant inter-group differences in the DCRs were observed (NLR < 5 vs. NLR > 5, *P* = 0.001, PLR < 135 vs. PLR > 135, *P* = 0.011) (Table 2). Patients with MLR < 0.31, NLR < 5, and PLR < 135 had a better DCR at 12 weeks than those with MLR > 0.31, NLR < 5, and PLR < 135, respectively (*P* < 0.05; Figure 1).

**TABLE 2:** Statistical associations between MLR, PLR, NLR, and treatment response.

	CR + PR (*n* = 14, 12.61%)	PD + SD (*n* = 97, 87.39%)	*P* value	CR + PR + SD (*n* = 74, 66.66%)	PR (*n* = 37, 33.34%)	*P* value
MLR < 0.31 (*n* = 61)	11	50	0.057	49	12	**0.001**
MLR > 0.31 (*n* = 50)	3	47		25	25	
PLR < 135 (*n* = 55)	8	47	0.543	43	12	**0.011**
PLR > 135 (*n* = 56)	6	50		31	25	
NLR < 5 (*n* = 94)	12	82	0.909	69	25	**0.001**
NLR > 5 (*n* = 17)	2	15		5	12	

**P* values in bold indicate statistically significant differences (*P* < 0.05).*

*CR, complete response; MLR, monocyte-to-lymphocyte ratio; NLR, neutrophil-to-lymphocyte ratio; PLR, platelet-to-lymphocyte ratio; PR, partial response; SD, stable disease.*

### Association Between Patient Variables and PFS and OS

Uni- and multivariate analyses were carried out to identify variables associated with PFS and OS. As of October 26, 2020, 77 patients (69.37%) showed disease progression. In the univariate Cox regression analysis, there were no significant differences in PFS with regard to sex, age, and combination with chemotherapy/radiotherapy/targeted therapy; however, baseline MLR, NLR, PLR, BMI, treatment line, and liver metastasis were associated with PFS (*P* < 0.05; Table 3). The multivariate Cox analysis indicated that MLR, treatment line, and BMI were factors associated with PFS.

**TABLE 3:** Uni- and multivariate analyses of PFS and OS.

	PFS				OS			
		
	Univariate		Multivariate		Univariate		Multivariate	
				
	HR (95% CI)	*P* value	HR (95% CI)	*P* value	HR (95% CI)	*P* value	HR (95% CI)	*P* value
Sex (male vs. female)	0.845 (0.536–1.332)	0.469	–	–	0.532 (0.290–0.987)	0.042	0.392 (0.210-0.731)	0.003
Age (≤65 vs. >65 years)	1.114 (0.650–1.910)	0.659	–	–	1.649 (0.763–3.566)	0.204	–	–
Treatment line (first vs. >first)	1.939 (1.228–3.064)	0.005	1.639 (1.018–2.639)	0.042	2.109 (1.146–3.882)	0.016	–	–
BMI (< 18.5 vs. ≥18.5)	0.246 (0.129–0.471)	0.001	2.074 (1.043–4.127)	0.025	0.159 (0.780–0.326)	(0.001	0.176 (0.082–0.377)	<0.001
MLR (<0.31 vs. ≥0.31)	2.405 (1.506–3.839)	0.001	1.813 (1.076–3.055)	0.025	2.630 (1.437–4.814)	0.001	2.184 (1.140–4.183)	0.018
PLR (<135 vs. ≥135)	1.184 (1.196–2.968)	0.006	–	–	1.768 (0.966–3.235)	0.065	–	–
NLR (< 5.0 vs. 5.0)	3.425 (1.951–6.014)	(0.001	1.887 (0.968–3.677)	0.062	2.343 (1.155–4.753)	0.015	–	–
Liver metastasis (no vs. yes)	1.648 (1.047–2.595)	0.031	–	–	1.525 (0.850–2.739)	0.154	–	–
Chemotherapy (yes vs. no)	1.529 (0.959–2.438)	0.074	–	–	1.233 (0.662–2.296)	0.509	–	–
Targeted therapy (yes vs. no)	1.343 (0.847–2.132)	0.210	–	–	1.091 (0.602–1.978)	0.774	–	–
Radiotherapy (yes vs. no)	1.044 (0.421-2.588)	0.926	–	–	1.232 (0.381-3.989)	0.727	–	–

**P* values in bold indicate statistically significant differences (*P* < 0.05).*

*BMI, body mass index; CI, confidence interval; HR, hazard ratio; MLR, monocyte-to-lymphocyte ratio; NLR, neutrophil-to-lymphocyte ratio; PLR, platelet-to-lymphocyte ratio.*

**FIGURE 2:**
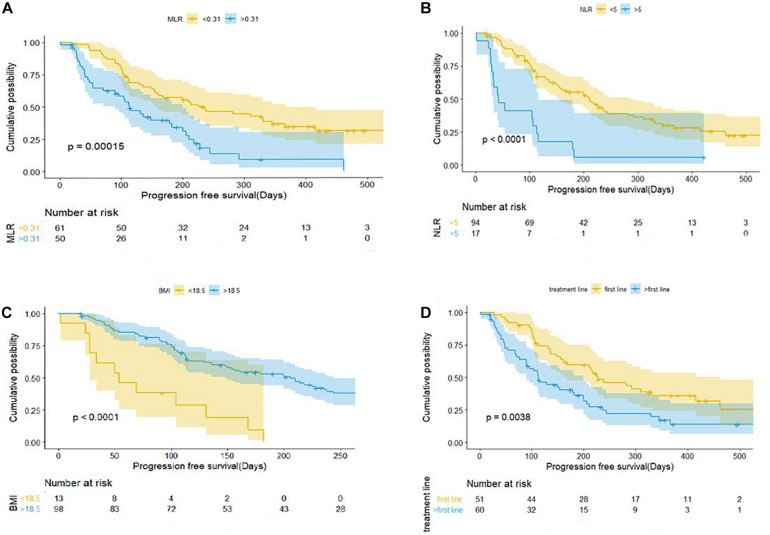
Kaplan–Meier curves of progression-free survival for patients with gastric cancer and colorectal cancer in relation to baseline clinical parameters. **(A)** MLR. **(B)** NLR. **(C)** BMI. **(D)** Number of treatment lines. BMI, body mass index; MLR, monocyte-to-lymphocyte ratio; NLR, neutrophil-to-lymphocyte ratio.

**FIGURE 3:**
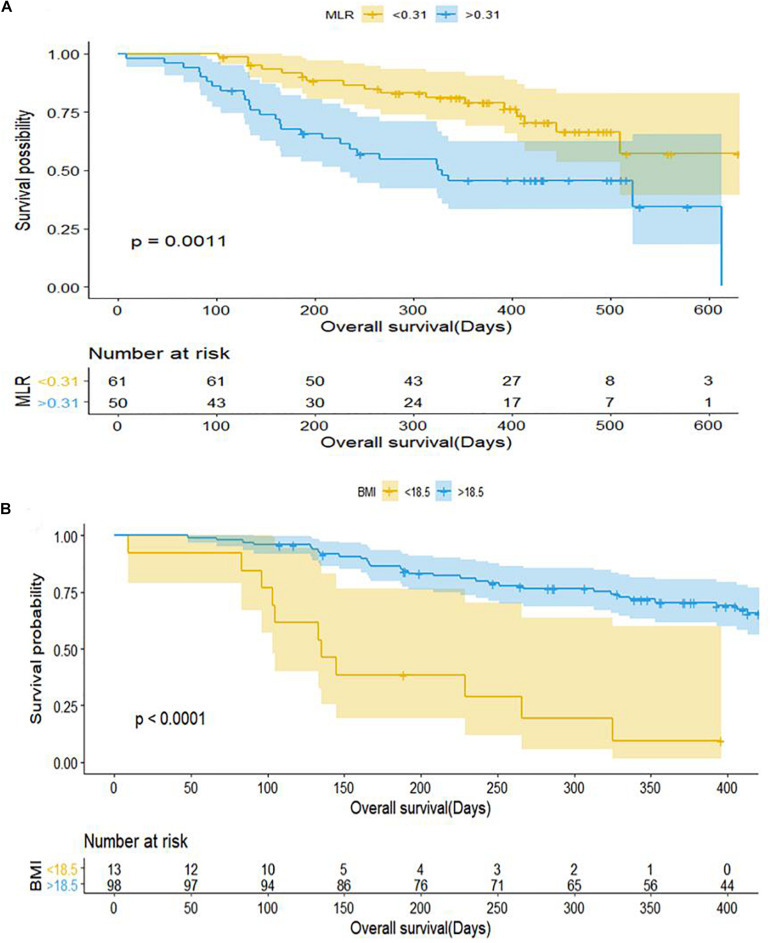
Kaplan–Meier curves of overall survival for patients with gastric cancer and colorectal cancer in relation to baseline clinical parameters. **(A)** MLR. **(B)** BMI. BMI, body mass index; MLR, monocyte-to-lymphocyte ratio.

Patients with baseline MLR < 0.31 had a longer PFS than those with MLR > 0.31 (mPFS, 7.5 months, 95% CI: 4.328–10.732 vs. 3.767 months, 95% CI: 2.635–4.899; *P* < 0.001) (Figure 2A). Patients with NLR < 5 had a significantly shorter PFS than those with NLR > 5 (mPFS, 7.0 months, 95% CI: 5.083–8.917 vs. 1.4 months, 95% CI: 0.504–2.296; *P* < 0.001) (Figure 2B). PFS was shorter in patients with BMI < 18.5 compared to those with BMI > 18.5 (mPFS, 1.80 months, 95% CI: 0.430–3.170 vs. 6.867 months, 95% CI: 4.924–8.810; *P* < 0.001) (Figure 2C). Patients who received anti–PD-1 inhibitor in the first-line setting had a longer PFS than those who had received prior treatments (mPFS, 7.60 months, 95% CI: 4.831–10.369 vs. 3.767 months, 95% CI: 2.770–4.763; *P* < 0.001) (Figure 2D). Pretreatment MLR (HR = 2.184, 95% CI: 1.140–4.183; *P* = 0.018) and BMI (HR = 0.176, 95%CI: 0.082–0.377; *P* < 0.001) were independent prognostic factor for OS (Figure 3).

### Correlation Between Inflammatory Markers and irAEs

**FIGURE 4:**
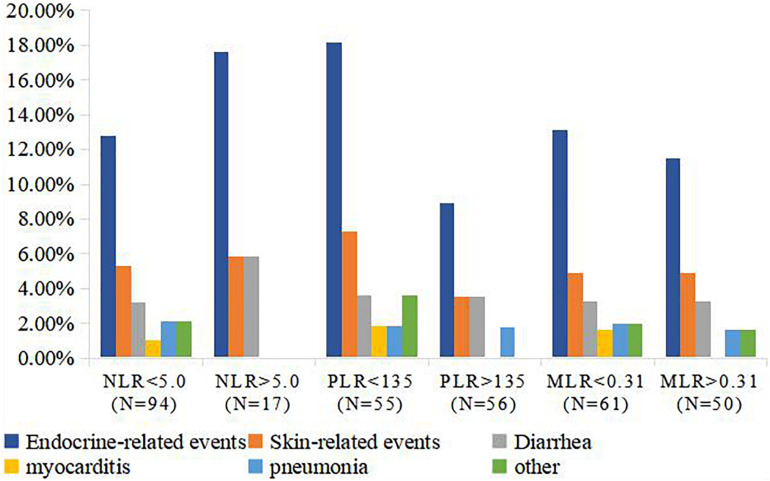
Rate of irAEs in NLR, MLR, and PLR groups. *P* values in bold indicate statistically significant differences (*P* < 0.05). irAE, immune-related adverse event; MLR, monocyte-to-lymphocyte ratio; NLR, neutrophil-to-lymphocyte ratio; PLR, platelet-to-lymphocyte ratio.

The majority of irAEs in our patient population were grade I, or II. A total of 30 patients (27.00%) had irAEs, including 15 (50.00%) endocrine-related, 6 (20.00%) skin-related, 4 (13.33%) diarrhea, 1 (3.33%) myocarditis, and 2 (6.66%) pneumonia events, and 2 (6.66%) cases of other events. The details and rate of irAEs were shown in Figure 4. The rate of irAEs was higher in the low PLR (<135) group compared to the high PLR (>135) group (36.36% vs. 17.85%; *P* = 0.028). There were no significant associations between MLR and NLR and risk of irAE; irAE rates were 26.93 and 27.11% in the high (>0.31) and low (<0.31) MLR groups, respectively (*P* = 0.810); and 26.60 and 29.41% in the high (>5) and low (<5) NLR groups, respectively (*P* = 0.107; Table 4).

**TABLE 4:** Statistical associations between MLR, PLR, NLR, and irAEs.

Characteristic	NLR		*P* value	PLR		*P* value	MLR		*P* value
					
	NLR < 5 (*N* = 94, %)	NLR > 5 (*N* = 17, %)		PLR < 135 (*N* = 55, %)	PLR >135 (*N* = 56, %)		MLR <0.31 (*N* = 59, %)	MLR >0.31 (*N* = 52, %)	
irAEs			0.810			0.028			0.107
Yes	25 (26.60)	5 (29.41)		20 (36.36)	10 (17.86)		16 (27.12)	14 (26.92)	
No	69 (73.40)	12 (70.59)		35 (63.64)	46 (82.14)		43 (72.88)	38 (73.08)	

**P* values in bold indicate statistically significant differences (*P* < 0.05).*

*MLR, monocyte-to-lymphocyte ratio; NLR, neutrophil-to-lymphocyte ratio; PLR, platelet-to-lymphocyte ratio; irAEs, immune-related adverse events.*

### Subgroup Analysis of the Relationship Between Inflammatory Indicators and PFS

**FIGURE 5:**
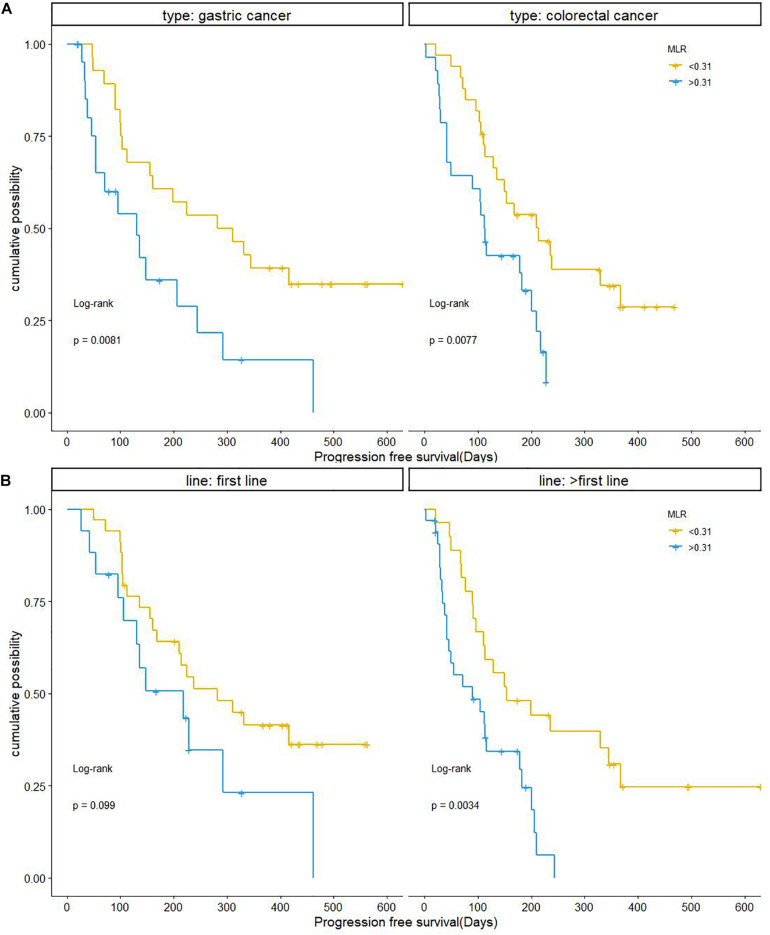
Kaplan–Meier curves of progression-free survival in relation to baseline MLR according to cancer type and number of treatment lines. **(A)** GC and CRC. **(B)** Number of treatment lines. CRC, colorectal cancer; GC, gastric cancer; MLR, monocyte-to-lymphocyte ratio.

Monocyte-to-lymphocyte ratio showed the same association with PFS in patients with GC and CRC who were treated with anti-PD-1 therapy. Compared to the MLR > 0.31 group, patients with MLR < 0.31 had a significantly longer PFS in both GC and CRC. PFS was shorter in GC patients with MLR > 0.31 compared to those with MLR < 0.31 (mPFS, 4.367 months, 95% CI: 1.632–7.102 vs. 9.4 months, 95% CI: 3.696–15.104; *P* = 0.0081); and was shorter in CRC patients with MLR > 0.31 than in those with MLR < 0.31 (mPFS, 3.733 months, 95% CI: 3.225–4.241 vs. 7.133 months, 95% CI: 3.750–10.516; *P* = 0.0077) (Figure 5A).

In patients who received anti-PD-1 therapy as second-line or later therapy, MLR < 0.31 was a predictor of longer PFS (mPFS, 5.113 months, 95% CI: 1.282–8.984 vs. 3.00 months, 95% CI: 0.462–5.538; *P* = 0.0034). However, MLR had no obvious predictive value for PFS in patients receiving anti–PD-1 inhibitor as first-line treatment. The mPFS in patients with MLR > 0.31 was 7.267 months (95% CI: 2.284–12.250), while the mPFS in patients with MLR < 0.31 was 9.40 months (95% CI: 5.098–13.702, *P* = 0.099; Figure 5B).

## Discussion

Despite the widespread application of immunotherapy in cancer treatment, there is a lack of biomarkers that can be used to evaluate therapeutic response and predict prognosis. At present, the evaluation indexes of immunotherapy are motley. It is reported that highly aneuploid tumors exhibit inherent resistance to anti-PD-1 therapy, which is associated with reduced the expression of genes specific for cytotoxic activities mediated by CD8 + T cells and altered genes in pathways related to ongoing immune response and microenvironment (Davoli et al., 2017). At the genetic level, tumor mutation burden (Cao et al., 2019) and alterations in DNA damage response and repair genes including ATM, ERCC2, BRAC-2, FANCA, MSH6, and POLE can to some extent predict the response of ICIs in specific cancer types (Teo et al., 2018; Fares et al., 2019). However, response rates to anti–PD-1 treatment can differ between tumors with a similar mutation burden, suggesting that other mechanisms play an important role (Riemann et al., 2019). Programmed death ligand-1 (PD-L1) expression in tumor tissue is the most relevant biomarker for gauging the efficacy of anti–PD-1 axis inhibitor therapy. There are no guidelines on the use of PD-L1 expression to predict response to immunotherapy in CRC, nor is there definitive evidence for the significance of PD-L1 expression in CRC (Shitara et al., 2020). The inefficiency, inconvenience and high cost of these methods make them impracticable for large-scale clinical application.

In the present study, we conducted a retrospective analysis of the association between peripheral blood inflammatory markers and clinical response to anti-PD-1 therapy in patients with advanced GC and CRC. Our findings highlight the prognostic value of NLR, PLR, and MLR for both cancer types. We also demonstrated a correlation between NLR, MLR, PLR, and irAEs, which has not been previously reported for GC and CRC immunotherapy.

In our study, MLR was found to be a biomarker for efficacy of DCR, and an independent prognostic factor for PFS and OS in GC and CRC patients who received anti–PD-1 therapy. Currently, the mechanisms underlying the efficacy of anti–PD-1 treatment by inflammatory indicators are not very explicit. On the one side, MLR values in peripheral circulating blood were significantly associated with prognosis in both metastatic GC and CRC (Basile et al., 2020; Zhou et al., 2020). On the other side, myeloid-derived suppressor cells (MDSCs) are a hallmark of tumor-associated inflammation that mediate the suppression of T cell responses in lymphomas (Raber et al., 2014). MDSCs are a heterogeneous population of cells at different stages of differentiation, and can be divided into polymorphonuclear and monocyte MDSCs, which are morphologically and phenotypically similar to neutrophils and monocytes, respectively (Wu et al., 2020). It was well documented that certain chemokines such as CCL2 and CSF-1 or CXCR2 ligands promoted the recruitment of MDSCs from the circulation to the tumor microenvironment (Abrams, 2020). Liu et al. (2019) found that accumulation of monocyte MDSCs leaded to decreased tumor infiltrating lymphocytes (TIL) and increased tumorigenicity, aggravating immunosuppression. Moreover, there is accumulating evidence that elevated lymphocyte counts are negatively correlated with tumor proliferation and invasion; CD4^+^ T cells mediate long-term response to anti–PD-1 therapy and antagonize acquired resistance (Liang et al., 2016; Kagamu et al., 2020). PD-1 predominantly regulates effector T cell activity within tissue and tumors, and ICIs enhance anti-tumor immunity by blocking negative regulators of T cell functions (Pardoll, 2012). Therefore, MLR, the ratio of two cells, can reflect the state of systemic inflammation and tumor microenvironment to a certain extent. However, it remains to be determined whether the efficacy of these agents can be enhanced by eliminating MDSC, which provides new insights for future research.

Neutrophil-to-lymphocyte ratio with cutoff of five is one of the most frequently reported peripheral blood inflammatory factor with efficacy or prognostic value for patients receiving anti-PD-1therapy in advanced urothelial and hepatocellular carcinoma, non-small cell lung cancer, head and neck cancer and Melanoma (Bartlett et al., 2020; Dharmapuri et al., 2020; Nassar et al., 2020; Peng et al., 2020; Ueda et al., 2020). Previous studies have shown that the increase of NLR value is positively correlated with the proportion of combined positive score of PD-L1 < 1 and the decrease of tumor infiltrating lymphocytes (Franz et al., 2020). In present study, NLR is only a factor affecting PFS and OS in univariate analysis, but it has no significant prognostic effect in multivariate analysis. It may be that different tumor and stage leads to different level of NLR value or different cutoff value due to different inflammatory condition.

Platelets induce epithelial-to-mesenchymal migration of circulating tumor cells and promote tumor cell extravasation and metastasis (Schumacher et al., 2013; Copija et al., 2020). It is reported high NLR and PLR are associated with poor outcome and are useful predictors of the efficacy of anti–PD-1 therapy in many cancers (Dharmapuri et al., 2020; Kartolo et al., 2020). In this study, we found that PLR only was the factor influencing PFS in univariate analysis for patients treated with anti-PD-1 therapy in GC and CRC. Interestingly, PLR < 135 was associated with a higher probability of irAEs (*P* = 0.028); this is the first study reporting a correlation between blood levels of an immune indicator and risk of irAEs in GC immunotherapy. It was previously demonstrated that PLR was significantly associated with immunotherapy in NSCLC (Pavan et al., 2019). This discovery may be used as a convenient way to identify irAEs timely, which is essential for improving quality of life and reducing the costs of treatment. We did not observe any correlation between NLR, MLR, and irAEs, although this may require validation in a larger cohort.

**FIGURE 6:**
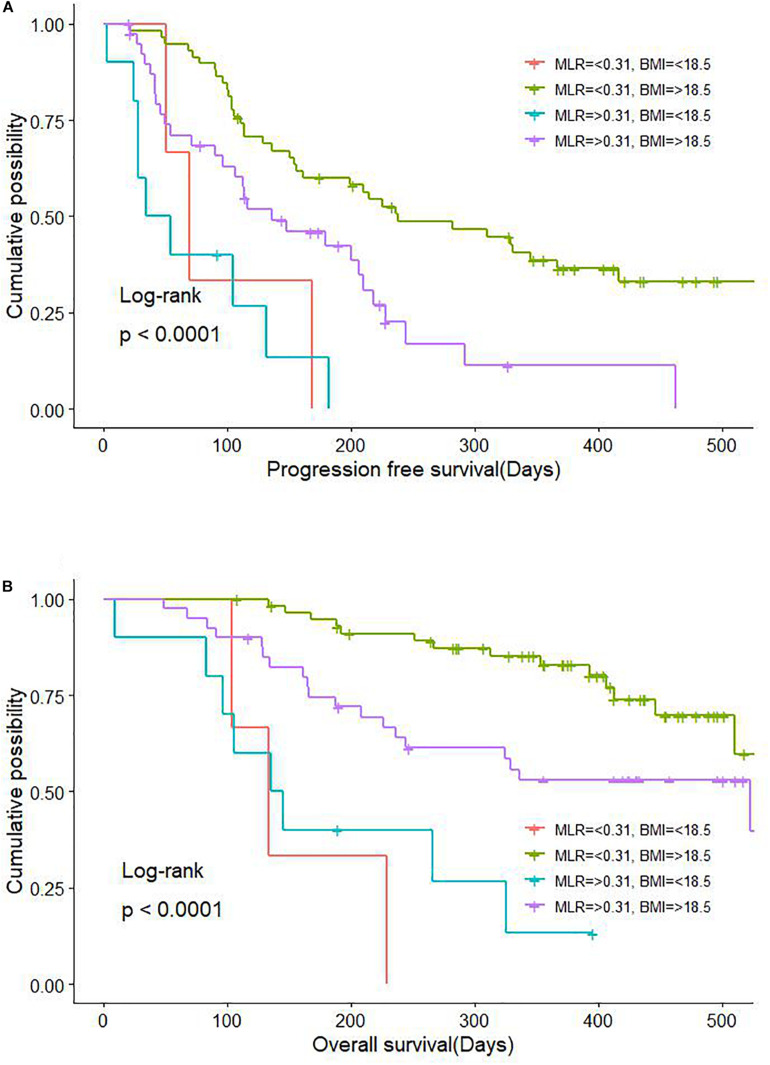
Combinatorial effect of MLR and BMI on survival in GC and CRC. **(A)** Effect on PFS. **(B)** Effect on OS. BMI, body mass index; CRC, colorectal cancer; GC, gastric cancer; MLR, monocyte-to-lymphocyte ratio; PFS, progression-free survival; OS, overall survival.

Body mass index predicted PFS in the Cox multivariate analysis. Marasmus (lower-than-normal BMI) was also a key factor influencing PFS in patients with GC. It was previously reported that BMI is associated with long-term survival and immunotherapy efficacy in patients with NSCLC, melanoma, and renal cancer (McQuade et al., 2018; Cortellini et al., 2019; De Giorgi et al., 2019) we observed that BMI > 18.5 was beneficial for both PFS and OS in patients with advanced GC receiving anti–PD-1 treatment. Moreover, the combination of BMI > 18.5 and MLR < 0.31 was associated with significantly longer PFS and OS (Figure 6). Patients with advanced GC and CRC are more likely to have a lower body fat percentage than those with non-gastrointestinal tumors, suggesting that our patients with cachexia (underweight) and high levels of inflammatory factors in the blood may not benefit from immunotherapy, which is an important consideration for treatment selection.

In the subgroup analyses of GC and CRC patients, a higher MLR value was negatively correlated with shorter PFS. We also observed a shorter PFS with MLR > 0.31 in patients who received ICI therapy as second- or later-line treatment, which was in accordance with the overall trend. In patients who received ICIs as first-line treatment, MLR < 0.31 showed a tendency toward longer PFS, although this lacked statistical significance(*P* > 0.05).

Besides the small sample size, limitations of the present study were the retrospective design and the fact that the data were collected at a single institution. The precise mechanism underlying the relationship between immune markers and treatment response also requires clarification. Finally, it remains unclear whether inflammatory marker levels are associated with MSI status and PD-L1 expression.

In conclusion, the results of this study demonstrate that peripheral blood inflammatory markers can serve as predictors of treatment response and prognosis in patients with advanced GC and CRC receiving anti–PD-1 therapy. MLR, NLR, and PLR were significantly correlated with DCR; MLR, and BMI were significantly independent factors for PFS and OS. Additionally, PLR < 135 may indicate an increased risk of irAEs. These findings can guide the selection and optimization of ICI regimens for patients with advanced GC and CRC, which can lead to better therapeutic outcomes. Thus, our work can serve as a valuable reference for treatment decisions in clinical practice.

## Data Availability Statement

The raw data supporting the conclusions of this article will be made available by the authors, without undue reservation.

## Ethics Statement

The studies involving human participants were reviewed and approved by Board of the Cancer Hospital of Harbin Medical University. The patients/participants provided their written informed consent to participate in this study. Written informed consent was obtained from the individual(s) for the publication of any potentially identifiable images or data included in this article.

## Author Contributions

XF and DW conceived the study, performed the data analysis, and wrote the manuscript. CL, ZM, QL, CL, and XL provided the technical guidance. HL, XG, WZ, JL, YB, and JY carried out the data collection. ZL and GW finalized the research results and final version of the manuscript. All authors contributed to the article and approved the submitted version.

## Conflict of Interest

The authors declare that the research was conducted in the absence of any commercial or financial relationships that could be construed as a potential conflict of interest.
